# Functional near-infrared spectroscopy and vagus somatosensory evoked potentials add to the power of established parameters such as poor cognitive performance, dsyosmia and APOe genotype to predict cognitive decline over 8 years in the elderly

**DOI:** 10.1007/s00702-024-02859-y

**Published:** 2024-11-13

**Authors:** Martin J. Herrmann, Alexandra Wuttke, Linda Breuninger, Judith Eff, Sophia Ettlinger, Matthias Fischer, Andrea Götzelmann, Annika Gram, Laura D. Pomper, Evelyn Schneider, Lisa Schwitalla, Niklas Siminski, Fabian Spielmann, Erik Weinmann, Viona Weyel, Julia B. M. Zeller, Martin Lauer, Jürgen Deckert, Thomas Polak

**Affiliations:** 1 Department of Psychiatry, Psychosomatics and Psychotherapy, Center of Mental Health, University Hospital Würzburg, Margarete-Höppel-Platz 1, D-97080 Würzburg, Germany; 2 SigmaCenter, Weihermatten 1, D-79713 Bad Säckingen, Germany; 3 Counselling Service, Johann Wolfgang Goethe University, Bockenheimer Landstraße 1334, D-60325 Frankfurt, Germany

**Keywords:** Prediction of cognitive impairments, VSEP, NIRS

## Abstract

Alzheimer’s dementia is the main cause of cognitive impairment in people over the age of 65, with Alzheimer’s disease starting presumably 10–15 years before the onset of clinical symptoms. It is therefore important to recognize dementia at an early stage and identify possible predictors. The existing methods, like different parameters of ß-Amyloid and Tau quantification in cerebrospinal fluid (CSF) or the living brain by measure of PET, are invasive and expensive. Therefore, the present study investigates the predictive value of a battery of clinical, neuropsychological, and blood parameters as well as two neurophysiological methods (functional near-infrared spectroscopy [fNIRS] and vagus somatosensory evoked potentials [VSEP]) which are easy to perform, less invasive and cost-efficient, for developing cognitive impairments in the elderly.

In this longitudinal, prospective study, we enrolled 604 healthy participants between 70 and 77 years of age. The participants were invited back after a mean time interval of 3 years and 11 months, and after 7 years and 8 months, and their cognitive impairments were determined.

Here we show that the development of cognitive impairments after approximately 8 years can be predicted not only by previously known risk factors such as ApoE4 risk alleles, dysosmia, or poor cognitive performance at baseline but that latency prolongation in the VSEP and altered functional activation patterns measured by NIRS at baseline also provide additional predictive value.

We therefore suggest that both neurophysiological parameters, VSEP and NIRS, should be included in future studies, investigating the prediction of dementia.

**Dementia ClinicalTrials.gov Identifier:** NCT02224326.

## Introduction

Due to the increasing lifespan of our world´s population, the prevalence of dementia is rising (Abbott 2011) and will even triple worldwide in the decades until 2050 (Collaborators 2022). Alzheimer´s disease (AD) as the most frequent cause of dementia (Alzheimer´s Association 2022) is until today still not yet curable, and it is also not possible to stop the disease (Scheltens et al. 2021). Therefore AD is of likewise increasing medical and socio-economic importance (Knopman et al. 2021) as it is also expressed by the proclamation of the “Decade of healthy aging brain 2021–2030”, declared by the United Nation´s general assembly (Chen et al. 2022).

The pathogenesis of AD is multifactorial in origin, and neural alterations are present at nearly every cellular level (Duchesne et al. 2024), comprising disturbance in production, accumulation, or removal of ß-Amyloid and its different precursors (Granzotto and Sensi 2024), regulation in Tau (Roda et al. 2022) and many other factors. Till today, more than 70 genetic risk factors have been identified in AD, the encoded proteins modulating or inducing the aforementioned processes (Bellenguez et al. 2022).

These pathogenic changes result in specific therapeutic options currently investigated in clinical trials (Sierksma et al. 2020). Most advanced are new disease modifying therapies (Perneczky et al. 2024) such as the Amyloid-targeting antibodies Gantenerumab, Donanemab, Aducanumab and Lecanemab (Menegaz de Almeida et al. 2024; Beshir et al. 2022), in part already granted accelerated and full approval by the U.S. Food and Drug Administration even if interpretation of the underlying studies and approval have been questioned (Høilund-Carlsen et al. 2024), among others because of clinical improvements of small effect sizes as opposed to increases in risk for amyloid-related imaging abnormalities (ARIA), rated as clinically meaningful (Ebell et al. 2024). Thus, disease modifying approaches only apply to a limited subset of patients in the AD continuum and against the background of several AD mimickers requiring stringent eligibility criteria (Masurkar et al. 2024), and they require application in early stages when treatment is most effective and less harmful. Thus, accurate and early diagnosis is crucial (Devi 2023).

Regardless of the specific pathogenic factor or therapeutic intervention to be stressed, many of the different pathogenic changes such as the formation of amyloid plaques, neurofibrillary tangles, or its precursors develop over a long time (Braak and Del Tredici 2015a; Braak and Tredici 2015b; Braak et al. 2011a; Cho et al. 2016; Eisele and Duyckaerts 2016) which calls for an early initiation of therapeutic interventions (Levey et al. 2006) when the irreversible damage is still minimal (Emery 2011; Gauthier 2005; Kasper 2012; Mangialasche et al. 2010; Palmer 2011). Thus, an early, preclinical diagnosis of developing AD is of utmost importance (Nestor et al. 2004).

Concerning biomarkers for an early diagnosis of AD, early longitudinal studies examined imaging (Noel-Storr et al. 2013; Saidlitz et al. 2014), cerebrospinal fluid (CSF) (Rosén et al. 2013), and blood (Lambert et al. 2013) including genetic (Moulder et al. 2013), proteomic (Ghidoni et al. 2013), and inflammatory parameters as well as neuropsychological and somatic biomarkers (Di Marco et al. 2014; Ellis et al. 2013) and are still ongoing (Iwatsubo 2022). As the mentioned early pathological features, however, are assumed to be mostly functional before resulting in irreversible morphological change, it is difficult to detect them with structural imaging methods (Linden and Thome 2011). Functional methods, instead, could be much more appropriate. As we know that functional change can occur even before behavioral or neuropsychological deficits (as it can be found for example for compensatory brain activity), we expect that the examination of functional brain activity with the advantage of a high temporal resolution during cognitive tasks will increase the predictive power of any assessment.

Prior studies have shown the predictive validity (sensitivity and specificity) of different cognitive measures for the development of AD such as associative learning (Ahmed et al. 2008) and verbal fluency (Clark et al. 2009). As such, tasks targeting verbal and non-verbal memory, visual-spatial and executive functions, and verbal fluency with an executive component seem to be suited for the investigation of the time course of cognitive deterioration (Reischies and Buerker 2005). Individuals developing AD were shown to perform worse on tests of episodic memory and semantic verbal fluency (Guarch et al. 2008) compared to persons not developing AD. It could be shown that composite cognitive test scores based on tests of episodic memory, executive function, and global cognition significantly predicted the development of AD (Rajan et al. 2015). Hence, an extended neuropsychological examination to measure these cognitive processes seems reasonable in particular to evaluate the additional benefit of other diagnostic methods (Snyder et al. 2014). The measurement of functional brain activity during or in addition to these tasks, however, may further improve their prognostic sensitivity because it is well-known that functional deficits can be compensated by additional brain activity, leading to normal behavioral performance (Grady 2012). In addition, functional changes in deep brain structures are known to occur many years before any cognitive deficit (Braak et al. 2011b). Thus, measuring functional brain activity has the potential to detect early changes in brain function, before neuropsychological deficits or even structural alterations can be detected.

In addition, it has been well known for a long time that today´s standard diagnostic repertoire such as exact morphometric measures, different parameters of ß-Amyloid and Tau quantification in CSF or in the living brain by the measure of PET can diagnose AD even before the onset of clinical symptoms (Bateman et al. 2012). These methods, however, are invasive, expensive, and/ or complex in the form of equipment. Therefore, a method to predict AD dementia should, if possible, be easy to perform, cost-efficient, non-invasive, and without any common criteria for exclusion, especially when it comes to examining people in preclinical stages who apparently do not yet have any symptoms of dementia in order to include them in studies, for example. An appropriate method for that purpose could be the use of electrophysiological methods (Bateman et al. 2012; Hoeppner et al. 2012; Muller et al. 1997; Pogarell et al. 2005) such as evoked potentials (Allison 1987; Juckel et al. 2008) or blood oxygenation-related parameters. Two such methods, one developed in our laboratory the other further developed, are functional near-infrared spectroscopy (fNIRS) and somatosensory evoked potentials of the vagus nerve (VSEP).

**fNIRS** makes it possible to measure the change in oxygen saturation of the cortex and thus to measure functional brain activity. In previous studies, it has been demonstrated that patients with mild cognitive impairments (MCI) show different functional activation to healthy controls when performing various tasks. Katzorke et al. (2018) were able to show that functional activation in the verbal fluency test is reduced in the inferior frontotemporal cortex in MCI patients. Another study by Haberstumpf and colleagues (Haberstumpf et al. 2022c) described that patients with MCI showed reduced activity in the partietal cortex during the clock-hand angle discrimination task. Cross-sectional analyses (Bonilauri et al. 2020) show that mild cognitive impairment (MCI) is associated with a change in functional activation, so it seems reasonable to investigate fNRIS as a potential early marker for future cognitive impairment.

The measurement of vagus somatosensory evoked potentials (**VSEP**) was first described by Fallgatter and colleagues (Fallgatter et al. 2003), and its significance was investigated in a number of studies in different clinical entities compared to healthy groups (Weise et al. 2015; Hagen et al. 2015; Polak et al. 2007, 2009, 2011, 2014; Metzger et al. 2012). A longer latency was observed for patients with cognitive decline (AD or MCI) (Polak et al. 2007), which was also found in patients with subjective memory impairment (SMI) (Hagen et al. 2015). In a follow-up study, patients with Alzheimer’s disease also showed latency prolongation (Metzger et al. 2012), while patients with MCI and healthy controls did not differ. In another study, longer latencies were found in AD compared to healthy controls, but there was no difference between major depression and healthy controls (Polak et al. 2014). These studies suggest that latency prolongation could be a potential marker for the development of AD.

The present data were collected as part of the prospective longitudinal observational study (Polak et al. 2017) to investigate the predictive value of a battery of clinical, neuropsychological, and blood parameters as well as the (additional) potential of fNIRS and VSEP measurements for the prediction of cognitive decline. The study hypothesizes that both deviating patterns of functional activation (fNIRS) and longer latencies (VSEP) provide additional predictive value for the development of cognitive decline.

## Methods

### The Vogel study

In 2011, we started a prospective, observational, long-term follow-up study, funded by the Vogel-Foundation Würzburg, intending to examine the predictive value of a battery of clinical, neuropsychological, and blood parameters as well as two electrophysiological examination parameters VSEP latencies and fNIRS activation patterns as non-invasive, convenient markers for the development of cognitive decline and AD dementia. The study is characterized by the following features: The participants come from a circumscribed recruitment area (Würzburg, Germany) and have a defined age at inclusion (70–77 years) to obtain a rather homogenous cohort. Exclusion criteria were set to minimize interference with diagnostic test procedures, such as Parkinson´s disease (affecting brainstem function similarly to AD), a recent history of stroke (confounding NIRS), and uncorrected hearing or vision impairment (potentially confounding neuropsychological testing). A total of *N* = 604 subjects were finally recruited and included in the study between June 2011 and October 2014. Each participant underwent a detailed examination, starting with a medical examination, neuropsychological assessments, measurements of functional brain activity in prefrontal and parietal brain areas using NIRS, and evoked potentials of the auricular branch of the Vagus nerve (VSEP). The design provided a follow-up for each participant after three and six years with careful examination of the cognitive state to evaluate if those who developed deterioration in the observation period already at baseline showed clinical, neurophysiological, laboratory or electrophysiological signatures different from those who remained cognitively stable (Polak et al. 2017).

### Participants

**Fig. 1:**
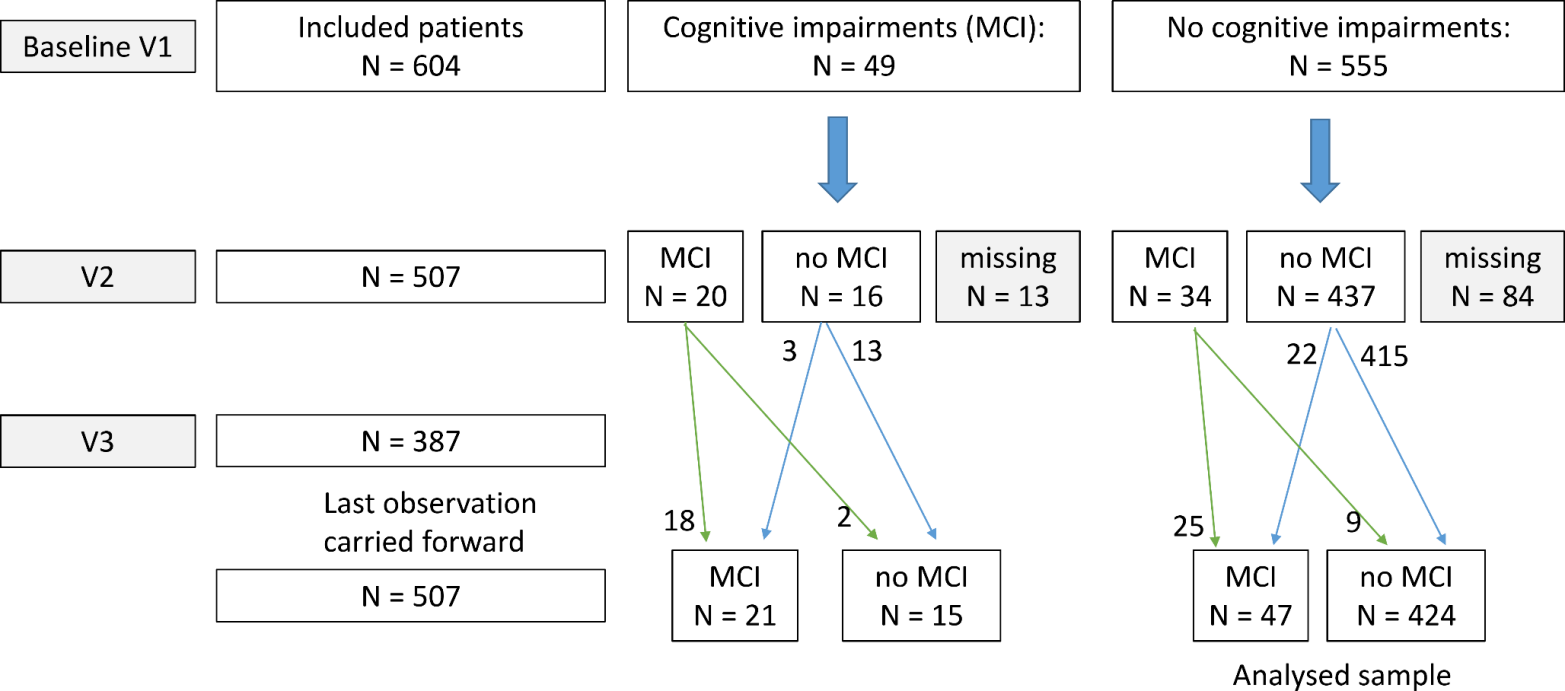
Chart flow of analyzed sample

At baseline (V1) Fig. 1, 604 participants (age between 70 and 77 years, *m* = 73.9, *sd* = 1.55) of both genders (313 males and 291 females) were included in the study (for details see: Polak et al. (2017). Due to the pandemic, there was a deviation from the original recall intervals (3 years and 6 years) in particular at V3. In contrast to previous analyses of the sample (Haberstumpf et al. 2020, 2022c), we did not exclude any participants at this stage. 507 participants could be assessed at visit 2 (V2) (mean time interval 3 years, 11 months), and 97 participants could not be reached for any reason. All 507 participants had a valid Mini-Mental State Examination (MMSE), DemTect was missing in 1 participant. At visit 3 (V3) (mean time interval 7 years, 8 months), 217 participants could not be reached, partial data are available for 387 participants. Out of the 387 participants at V3, MMSE data of 357, and DemTect data of 355 participants could be assessed.

We aimed to investigate the development of dementia first and described a MMSE sum score < 24 as our primary outcome (Polak et al. 2017). Only five participants fulfilled this criterion. Therefore, we focussed now on the development of cognitive impairments, with the definition of MMSE sum score < 27 or DemTect sum score < 13. Additionally, we applied a last observation carried forward approach for the MMSE and DemTect variables, which were missing at V3 but available at V2.

Based on this definition, 49 participants were impaired at V1, and 21 were still impaired at V2/V3 (58.3%). In the group of non-impaired participants at V1 (*N* = 555), 47 developed an impairment at V2/V3 (8.5%). This effect was highly significant (based on the reduced sample of *N* = 507 with *N* = 471 unimpaired and *N* = 36 impaired at V1; *Chi*^*2*^[1] = 67.3, *p* < 0.001). For further analyses, only participants who were non-impaired at V1 were included (*N* = 424 non-impaired and *N* = 47 impaired at V3). Study sample description and comparison between groups will be shown in the results section.

### Neuropsychology and questionnaires

The detailed neuropsychological assessments (see Polak et al. (2017) for details) were grouped into four factors using an exploratory factor analysis, as described before (Haberstumpf et al. 2022a, b). The individual factor loadings on the following four factors were further used for analyses: declarative memory, attention, working memory, and visual-spatial processing.

### *ApoE4* genotyping

As described before (Katzorke et al. 2017), *ApoE4* was genotyped according to Hixson and Vernier (1990). We used a polymerase chain reaction (PCR) with the forward primer 5’- TAA GCT TGG CAC GGC TGT CCA AGG A − 3’ and the reverse primer 5’- ACA GAA TTC GCC CCG GCC TGG TAC ACT GCC − 3’. In a volume of 25 µl the PCR mixture contained 2.5 µl Goldstar, 1 µl 25mM MgCl2, 1 µl 2.5mM Nuk each, 1 µl (10pmol/µl) of each primer, 0.3 µl Taq, 0.8 µl DMSO, 16.4 µl H_2_O and 50 ng of genomic DNA. The cycling conditions were 95 °C for five minutes, 45 s at 95 °C, 45 s at 65.2 °C and 45 s at 72 °C for 38 cycles, five minutes at 72° and a pause at 10 °C. The PCR product with a fragment size of 244 bp was digested using HinP1I (New England Biolabs, Frankfurt am Main, Germany) for two hours at 37 °C and then migrated on a peqGOLD MoSieve Agarose MS-500.

### NIRS

**Fig. 2:**
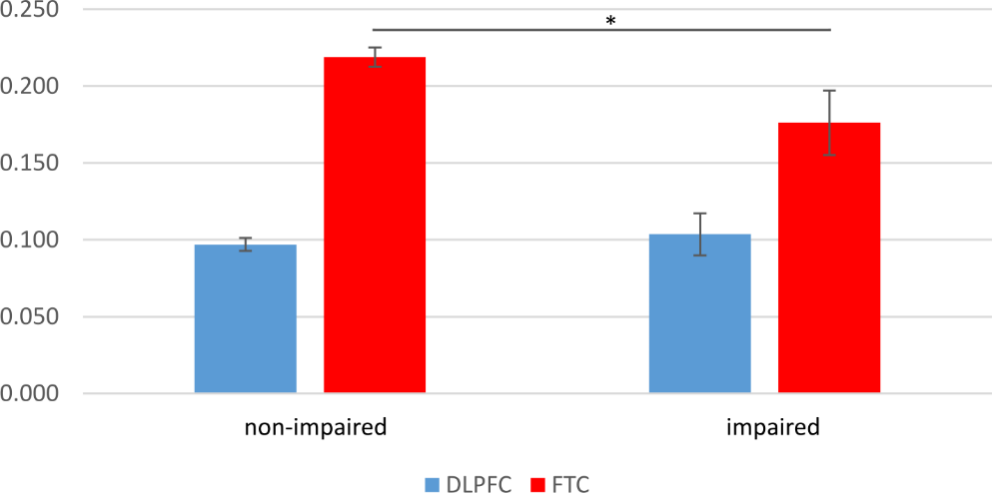
[O2Hb] of the letter version of the VFT during V1

We measured the changes in cerebral oxygenation with the Hitachi ETG-4000 NIRS system with 52 channels. The ETG-4000 uses near-infrared light with wavelengths between 650 and 850 nm and a sampling rate of 10 Hz. For the verbal fluency task (VFT, Katzorke et al. (2018), the 3 × 11 probe set was placed over the prefrontal cortex with the lowermost row over FPz according to the 10–20 system (see Fig. 2; American Electroencephalographic Society 1994). The VFT consisted of three conditions (letter, category, control task) lasting 30 s each, and were repeated three times with a 30-second rest between each active condition. Data were filtered with a cosine filter and a moving average with a time window of five seconds. After common average reference calculation, the effect sizes (activation period versus baseline) were calculated. For the angle discrimination test (ADT, Haberstumpf et al. (2020), we placed two probe sets (3 × 5) over the left and right parietal cortex (placed to the left and right of electrode position Pz). After filtering the data (moving average with five seconds, a band-pass filter of 0.08–0.5 Hz), we corrected the data for motion artifacts using a correlation-based method for signal enhancement developed by (Cui et al. 2010). Data were further analyzed with the general linear model (GLM) approach.

### Vagus somatosensory evoked potentials

VSEP were recorded bipolarly from electrode positions C3-F3, C4-F4, Fz-F3, Fz-F4, T3-O1, and T4-O2 and processed using a Medelec SynergyN™– EMG/EP Monitoring System (Viasys Healthcare, Höchberg, Germany). Electrode impedances were kept below 2 kΩ. As ground electrode, a band electrode was fixed around the head directly above the ears. Bipolar electrodes with very fine copper stranded wires at the end were used for stimulation according to Fallgatter and colleagues (Fallgatter et al. 2003), and were fixed with a small amount of Grass electrode paste (Grass Technologies, An Astro-Med, Inc. Subsidiary, West. Warwick, RI, USA) at the inner side of the tragus. The cathode and anode were approximately 5 mm apart. For stimulation, electrical square impulses of 0.1ms duration, 8 mA intensity, and a frequency of 0.5 Hz were applied at the left and right tragus in separate trials. The electrical brain activity was recorded with a sampling rate of 20 kHz, a band-pass of 0.1–1 kHz, and an epoch length of 10ms separately for right and left stimulation. 100 artifact-free epochs (artifact criterion ± 40 µV) per stimulation side were averaged. Only unambiguous potentials were included in the final data analysis (unique potential in the first 8 ms after stimulation; obligatory morphology with P1 as the first positive peak, followed by N 1 as the first negative peak and P2 as the second positive peak). Peaks were defined visually, and peak marks were set manually, latencies and amplitudes of the different VSEP components (latencies P1, N 1, and P2 as well as amplitudes P1N1 and N1P2) were then measured automatically.

## Results

### Baseline differences in basic variables

Table 1 shows that participants with impairments at V3 are less educated, with lower cognitive resources (DemTect and MMSE). Additionally, these participants already exhibit higher blood glucose concentrations and higher depression scores (GDS) at baseline.

**Table 1:** Study population description at baseline (visit 1)

		No impairments at V3 (*N* = 424)		Cognitive Impairments at V3 (*N* = 47)		Statistics		
		*M*	*SD*	*M*	*SD*	*t*	*df*	*p*
Age	in years	73.83	1.56	74.06	1.58	-0.98	469	0.330
Education	in years	14.64	3.90	13.13	2.91	2.58	465	**0.010**
BMI	in kg/m^2^	26.01	3.86	26.68	4.18	-1.11	466	0.269
RR in mmHg	Systolic	146.74	19.80	149.28	21.84	-0.82	468	0.411
	Diastolic	82.67	10.26	82.98	16.69	-0.18	468	0.856
Blood parameters	Cholesterol total (mg/dl)	215.52	41.42	208.60	37.43	1.10	469	0.273
	LDL (mg/dl)	128.15	36.48	121.38	35.72	1.21	469	0.227
	HDL (mg/dl)	64.84	18.32	61.64	15.86	1.15	469	0.250
	Homocysteine (micromole/l)	14.42	6.42	14.64	5.51	-0.23	466	0.822
	HbA1c (%)	5.76	0.49	5.91	0.74	-1.93	468	0.054
	Glucose (mg/dl)	95.15	17.03	103.77	28.40	-3.04	469	**0.003**
	Vitamin B12 (pg/ml)	450.1	243.3	520.9	376.7	-1.77	468	0.077
	Folic acid (ng/ml)	11.0	4.18	10.79	4.62	0.32	467	0.749
Cognitive parameters	DemTect	16.51	1.69	15.62	1.82	3.42	469	**0.001**
	MMSE	29.37	0.82	28.83	1.03	4.16	469	**0.001**
Affective parameters	BDI-II	6.25	5.54	7.57	6.62	-1.52	469	0.129
	GDS-15	1.39	1.90	2.17	2.22	-2.63	469	**0.009**
	ASI-3	16.95	14.15	18.83	12.50	-0.86	462	0.388
Daily Living	Bayer-ADL	1.43	0.59	1.53	0.70	-1.03	469	0.305

*M* Mean, *SD* Standard Deviation, BMI body mass index, RR arterial blood pressure, DemTect (Kalbe et al. 2004), MMSE (Folstein et al. 1975), GDS-15 (Sheikh and Yesavage 1986), ASI-3 (Reiss et al. 1986), Bayer-ADL (Hindmarch et al. 1998), BDI-II (Beck et al. 1996).

The gender distribution was equal in both groups (see Table 2). We found significant differences at V1 in distribution for dysosmia, *ApoE4* genotype, diagnosed diabetes mellitus, and the fact that relatives noticed memory problems in participants already at V1, but not in SMI. The lifetime prevalence of panic attacks tends to be different between both groups. In all of the mentioned significant variables, the percentage of developing cognitive impairments at V3 was higher in participants with positive results at V1 (dysosmia: yes 22.3%; no 7.1%; *ApoE4* risk alleles: yes 17.8%; no 7.9%; diabetes mellitus: yes 22.9%; no 8.5%; relatives notice memory impairments: yes 17.1%; no 8.5%; lifetime panic attacks: yes 15.8%; no 9.0%). The other variables assessed in this trial did not show significant differences between groups. This might partly also be explained by a small number of participants with traumatic brain injury (*N* = 4), smoking (*N* = 22), and previously increased and problematic alcohol consumption (*N* = 20).

**Table 2:** Study population description at baseline (visit 1) for dichotomy variables

		No impairments at V3	Cognitive impairments at V3	Chi^2^	*p*
Gender	Male	222	25	0.01	0.914
	Female	202	22		
Dysosmia	No	352	27	**18.03**	**0.001**
	Yes	66	19		
*ApoE4* risk gen	E3/E3	327	28	**8.56**	**0.008**
	E4/E4 or E3/E4	83	18		
Diabetes mellitus	No	386	36	**9.91**	**0.002**
	Yes	37	11		
Relatives notice memory impairments	No	355	33	**5.52**	**0.019**
	Yes	68	14		
Subjective Memory impairments (SMI)	No	148	15	0.17	0.683
	Yes	276	32		
Lifetime panic attacks	No	376	37	2.65	0.104
	Yes	48	9		

### Baseline differences in neuropsychological assessments

As already seen for the dementia screening instruments (see Table 1), participants with cognitive impairments at V3 also show cognitive deficits in the extended neuropsychological assessments in all the four extracted factors at V1 (see Table 3). Factor analysis was calculated for *N* = 590 participants. *N* = 543 were without any impairment at V1. For the comparison in Table 3, *N* = 417 non impaired, and *N* = 45 impaired participants could be included.

**Table 3:** Study population description at baseline (visit 1) for factors of cognitive performance

	No impairments at V3			Cognitive impairments at V3			Statistics			
	*M*		*SD*	*M*	*SD*		*t*	*df*		*p*
Declarative Memory	0.15	0.90		-0.47		1.02	4.30	460	**0.001**	
Working Memory	0.12	1.02		-0.31		0.85	2.78	460	**0.003**	
Visual-spatial processing	0.16	0.86		-0.48		1.36	4.46	460	**0.001**	
Attention	-0.07	1.01		0.25		0.97	-2.04	460	**0.021**	

Displayed are mean (M) and standard deviations (SD) for factor loading on the extracted four factors of neuropsychological assessments. Note, that factor attention is inverse to the other factor, for factor attention higher values indicate lower performance.

### Functional near-infrared spectroscopy (fNIRS)

We used the analysed NIRS data from our previous papers based on the baseline data (V1; Haberstumpf et al. 2020, 2022c; Katzorke et al. 2017, 2018; Zeller et al. 2019). For the **verbal fluency task** (VFT, Katzorke et al. (2018) we used the region of interests (ROI) of the dorsolateral prefrontal cortex (dlPFC) and the frontotemporal cortex (FTC) of the left and right hemisphere, for both chromophores (O2HB and HHB) for both versions of the VFT (category and letter, after contrasting with the control condition). Data from 8 participants are missing. For the VFT analysis, *N* = 417 non-impaired, and *N* = 46 impaired participants were included.

In the letter version we found for [HHB] a main effect region (*F*[1,461] = 107.7, *p* < 0.001), but no main effects group (*F*[1,461] = 0.1, *p* = 0.76), or hemisphere (*F*[1,461] = 0.51, *p* = 0.48). A tendency for hemisphere * group was found (*F*[1,461] = 2.99, *p* = 0.09). All other interactions were non-significant.

For [O2Hb] in the letter version we found again a main effect region (*F*[1,461] = 124.3, *p* < 0.001), but no main effects group (*F*[1,461] = 1.48, *p* = 0.22), or hemisphere (*F*[1,461] = 0.60, *p* = 0.44). Here the interaction region * group was highly significant (*F*[1,461] = 8.05, *p* < 0.001). All other interactions were non-significant.

Figure 2 shows the mean [O2Hb] values for both regions. Post-hoc t-test revealed reduced [O2Hb] in the FTC region at V1 for participants developing a cognitive impairment at V3 (*M* = 0.18 ± 0.14) compared to non-impaired at V3 (non-impaired: *M* = 0.22 ± 0.13; *t*[461] = 2.11, *p* < 0.05).

In the category version of the VFT we found only a main effects of region ([O2Hb]: *F*[1,461] = 53.5, *p* < 0.001; [HHb]: *F*[1,461] = 15.8, *p* < 0.001).

For the clock-hand-angle discrimination task (ADT, (Haberstumpf et al. 2020, 2022c), the data were corrected for motion artifacts (Cui et al. 2010) resulting in only one parameter, instead of [O2Hb] and [HHb]. Here we used the ROIs over the left and right parietal cortex. Data from 10 participants are missing. For the ADT analysis, *N* = 414 non-impaired, and *N* = 47 impaired participants at V3 were included. We found a main effect of pointer length (*F*[2,918] = 11.4, *p* < 0.001), laterality (*F*[2,918] = 10.8, *p* < 0.001), but no main effect of group (*F*[1,459] = 0.1, *p* = 0.76). The interaction laterality * group reached significance (*F*[2,459] = 10.0, *p* < 0.002). All other interactions were non-significant. For post-hoc tests, the mean values for the different pointer lengths and the differences between the hemispheres (right minus left) were calculated. Impaired participants at V3 show reduced right laterality (*M* = 0.0002 ± 0.020) at V1 compared to non-impaired participants (*M* = 0.012 ± 0.025; *t*[459] = 3.17, *p* < 0.002).

### Vagus somatosensory evoked potentials (VSEPs)

VSEPs were measured in *N* = 533 participants, but the number of valid data differed for electrode positions (valid data are available in maximal *N* = 380 participants: unimpaired participants at V1 *N* = 340, impaired at V1 *N* = 40). As described in Table 4, the latency for the P2 peak over T4O2 after right-sided stimulation is significantly prolonged at V1 in participants who will develop a cognitive impairment at V3.

**Table 4:** Latencies of the VSEP components over the different electrode positions

			No impairments at V3			Cognitive Impairments at V3			Statistics		
	Electrode positions	Peak	*M*	*SD*	*M*		*SD*	*t*		*df*	*p*
Right stimulation	C3F3	P1	2.37	0.87	2.33		0.87	0.25		340	0.804
	C3F3	N1	3.67	1.28	3.77		1.16	-0.39		340	0.697
	C3F3	P2	4.83	1.57	5.15		1.30	-1.11		339	0.269
	C4F4	P1	2.20	0.84	2.41		1.04	-1.42		373	0.156
	C4F4	N1	3.43	1.14	3.65		1.12	-1.10		373	0.272
	C4F4	P2	4.70	1.43	5.03		1.26	-1.37		373	0.172
	FzF3	P1	2.05	0.90	2.30		1.04	-1.62		369	0.105
	FzF3	N1	3.30	1.13	3.36		1.04	-0.32		369	0.752
	FzF3	P2	4.69	1.40	4.67		1.13	0.11		368	0.916
	FzF4	P1	2.34	1.33	2.39		0.83	-0.21		339	0.836
	FzF4	N1	3.70	1.33	3.94		1.39	-1.02		339	0.309
	FzF4	P2	4.93	1.52	5.30		1.78	-1.32		339	0.186
	T4O2	P1	1.82	0.90	2.01		1.20	-1.24		382	0.214
	T4O2	N1	3.15	0.98	3.52		1.25	-2.16		382	0.032
	**T4O2**	P2	**4.88**	**1.33**	**5.60**		**1.53**	**-3.20**		**382**	**0.00149**
Left stimulation	C3F3	P1	2.18	0.86	2.02		0.72	1.04		369	0.299
	C3F3	N1	3.49	1.21	3.51		1.26	-0.08		369	0.934
	C3F3	P2	4.78	1.56	4.74		1.51	0.13		369	0.897
	C4F4	P1	2.33	0.87	2.06		0.66	1.83		333	0.068
	C4F4	N1	3.65	1.26	3.21		0.93	2.07		333	0.039
	C4F4	P2	4.81	1.53	4.35		1.24	1.76		333	0.079
	FzF3	P1	2.13	0.95	2.21		0.99	-0.47		348	0.638
	FzF3	N1	3.46	1.44	3.66		1.51	-0.78		348	0.439
	FzF3	P2	4.62	1.65	4.84		1.90	-0.74		348	0.458
	FzF4	P1	2.03	0.86	1.75		0.60	1.93		375	0.055
	FzF4	N1	3.24	0.98	3.28		0.93	-0.27		375	0.789
	FzF4	P2	4.68	1.29	4.84		1.48	-0.71		375	0.478
	T3O1	P1	1.80	0.86	1.59		0.54	1.42		393	0.157
	T3O1	N1	3.22	0.98	3.14		1.29	0.48		394	0.630
	T3O1	P2	4.97	1.39	4.91		1.62	0.23		394	0.817

Corrected alpha 0.05 / 30 = 0.00167

### Prediction of cognitive decline

For the logistic regression, we included *N* = 347 participants (*N* = 311 non-impaired participants and *N* = 36 impaired patients) with valid data in all predictor variables (significant effects for group differences: education, Glucose, depressive symptoms (GDS), dementia screening scores (DemTect, MMSE), factor loadings of detailed neuropsychological assessments (declarative memory, working memory, visual-spatial processing, attention), the NIRS parameter of [O2HB] during the letter (minus control) condition of the FTC, the P2 peak latency of the VSEP over T4-O2 electrode position for right-sided stimulation, and the categorical variables: relatives mentioned memory problems, diabetes mellitus, dysosmia, *ApoE4* risk allele. For logistic regression, we z-normalized the non-categorial predictors based on the sample of healthy participants at V1 and without missing data for the classification at V3. The factor loadings declarative memory, working memory, and visual-spatial processing were inverted by multiplying with − 1 (so higher values indicate worse performance), for a better interpretation of the Odds ratio. For the calculation of the logistic regression, we used a backward exclusion of the parameters (*p* > 0.1). After seven steps the model was still significant (*Chi*^*2*^[10] = 85.26, *p* < 0.001, -2ll = 146,0; Cox & Snell R^2^ = 0.218; Nagelkerkes R^2^ = 0.448). The final model reaches an accuracy of 91.6%, with a specificity of 0.98 and a sensitivity of 0.33. The results show (Table 5) that the highest risk factor of our analyses is the presence of at least one *ApoE4* risk allele e4, followed by dysosmia, and the third-party report of memory deficits of the patients by relatives. Besides the objective measured cognitive deficits in the domain of declarative memory, visual-spatial processing, working memory, and attention, both neurophysiological parameters, prolonged VSEPs latencies, and functional brain activation during the ADT add additional predictive value to the cognitive status at V3.

**Table 5:** Parameters of the logistic regression predicting cognitive deficits at V3 based on the baseline variables at V1

					95% Confidence interval for Odds Ratio		
	*b*	se	Wald	*p*	lower	odds	upper
Constant	-4.27	0.50	72.86	0.001	-	0.01	-
*ApoE4* risk allele	1.89	0.53	12.83	0.001	2.35	6.61	18.57
Dysosmia	1.24	0.48	6.61	0.010	1.34	3.45	8.86
Relatives mentioned memory deficits	1.01	0.50	4.14	0.042	1.04	2.75	7.26
Declarative Memory deficits	0.88	0.24	13.05	0.001	1.49	2.40	3.87
VSEP latency	0.73	0.24	9.22	0.002	1.30	2.08	3.33
Visual-spatial processing deficits	0.71	0.26	7.70	0.006	1.23	2.04	3.38
Working Memory deficits	0.71	0.26	7.51	0.006	1.23	2.04	3.40
Left lateralization during ADT	0.66	0.29	5.19	0.023	1.10	1.94	3.44
Attention deficits	0.52	0.26	4.01	0.045	1.01	1.68	2.80
Glucose (mg/dl)	0.30	0.16	3.40	0.065	0.98	1.35	1.85

*b*: regression coefficient, *se*: standard error

**Fig. 3:**
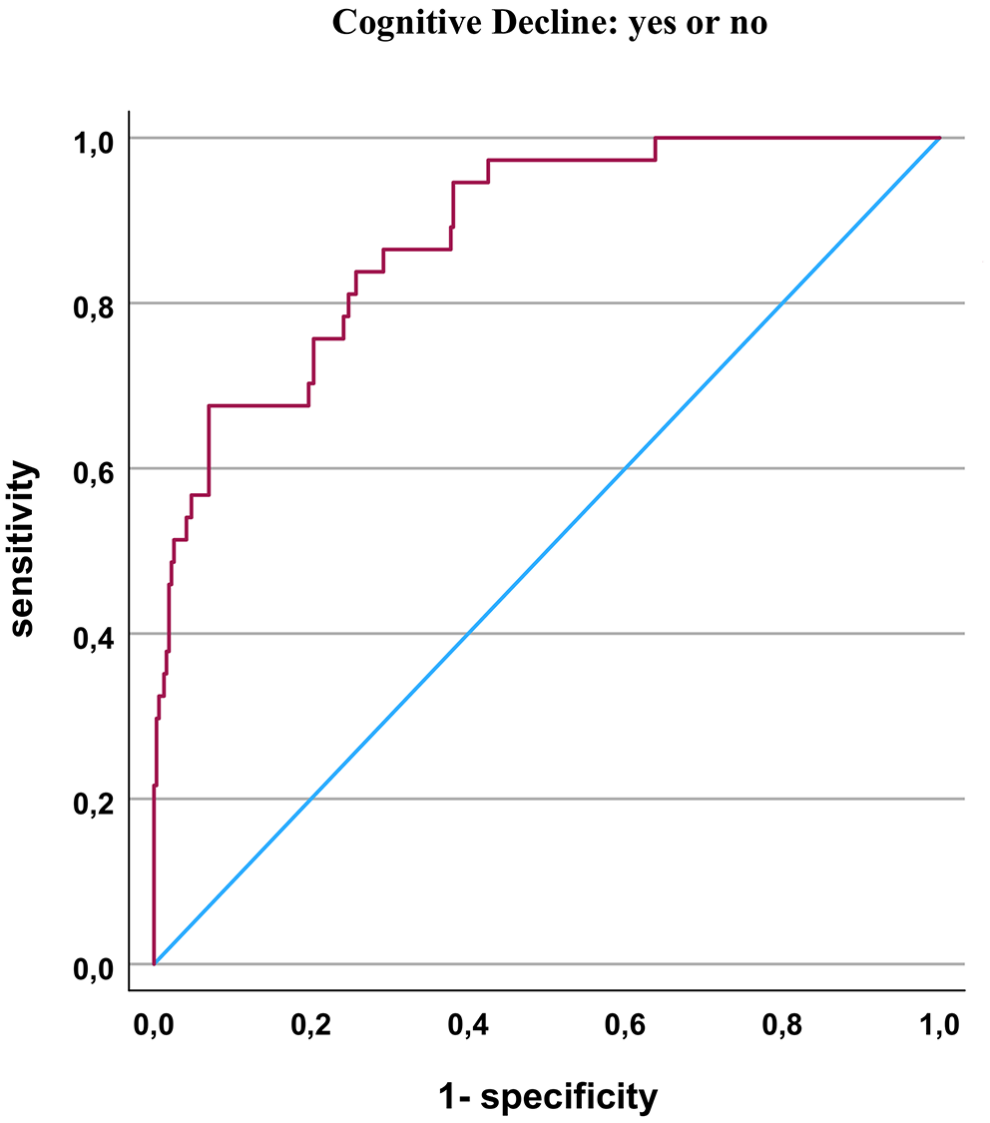
ROC-curves showing the predictive performance for cognitive decline from V1 to V3 (LOCF)

A receiver operating characteristic (ROC) analysis was performed to evaluate the diagnostic performance of the developed logistic regression model and to determine the optimal classification threshold. The significant predictors were summarized (weighted according to the odds ratio), and used as test variable for the dependent variable cognitive status at V3 as defined by LOCF (see Fig. 3). The AUC was 0.88. A sensitivity of 0.81 was reached at a specificity of 0.74 (for a value of 5.06 for the weighted sum score).

## Discussion

The aim of the Vogel study was to investigate the potential of a battery of clinical, neuropsychological, blood parameters, and as the main aim, the two neurophysiological examination methods functional near-infrared spectroscopy (fNIRS) and vagus somatosensory evoked potentials (VSEP) measurements for the prediction of cognitive decline in a prospective longitudinal observational study (Polak et al. 2017). For this purpose, we included 604 healthy participants aged between 70 and 77 years in this study, examined the participants in detail at the baseline measurement, and characterized them again after approximately 4 and 8 years.

The main finding of this study was that in addition to previously described, well known risk factors for cognitive decline in older age, such as *ApoE4* risk alleles (Livingston et al. 2020), a lack of cognitive reserve (Chen et al. 2022), and dysosmia (Fatuzzo et al. 2023) indicating the validity of our study´s results, both latency prolongation of the VSEP and a change in functional brain activation during a visual-spatial task also predicted the development of cognitive impairment.

The latency prolongation of **VSEP** would fit findings regarding the early involvement of brainstem nuclei (Attems et al. 2012; Braak et al. 2011a), and underscores previous studies showing VSEP specific latency effects. As such, prolongation of VSEP latencies was found in neurodegenerative diseases such as Alzheimer’s (Polak et al. 2007, 2014) and Parkinson´s disease (Polak et al. 2011) as well as and Subjective memory impairment (SMI; Hagen et al. 2015) as possible precursors opposed to Vascular dementia (Polak et al. 2009) and Major depression (Polak et al. 2014) without consistent brainstem affection. However, longitudinal studies on VSEP have not yet ever be done so far; therefore, the presented is the first study on longitudinal development and predictive validity of VSEP.

For the **NIRS** parameter, we found differences between participants with impairments and no impairments for the VFT as well as for the visual processing task ADT, confirming previous cross-sectional studies comparing MCI patients with healthy controls (Haberstumpf et al. 2022c; Katzorke et al. 2018). Here we also found differential effects years before a cognitive impairment to be detectable. In the prediction model, only the effect of functional brain activity during the visual-spatial processing survived multivariate analyses. This is even more relevant as the neuropsychological testing also included visual-spatial processing, which was also included in our predictive model. This underscores the additional value of the functional NIRS measurements of brain activity, indicating that compensatory brain activity might cover early cognitive deficits leading to normal behavioural performance (Grady 2012). As mentioned before, functional changes in deep brain structures are known to occur many years before any cognitive deficit (Braak et al. 2011b). Our study showed that measuring functional brain activity as well as VSEPs has the potential to detect early changes in brain function additionally to neuropsychological deficits.

Although we found that participants developing a cognitive impairment at V3 as defined by LOCF compared to participants staying cognitively unimpaired, were less educated (Livingston et al. 2020), had a higher blood glucose level with more often diagnosed diabetes mellitus (Li et al. 2016), had higher depression scores (Divers et al. 2022) and reduced performance in dementia screening instruments like MMSE or DemTect (Chen et al. 2022), these parameters do not add additional predictive value in a multivariate analysis with our detailed neuropsychological assessment and the electrophysiological parameters. This means that the predictive value of these non-significant variables can be better explained by the other parameters.

The incremental benefit of including objective markers such as fNIRS and VSEP in early dementia diagnosis has manifold advantages. They are particularly relevant given the often-observed anosognosia in people living with dementia (Wilson et al. 2016). Likewise, in this study, the predictive value of relatives observing memory difficulties was higher than that of patients’ self-report. The advantage of objective markers for dementia diagnosis thus might allow compensating anosognosia in people living with dementia and might relieve the tension and imbalance in the relationship between people living with dementia and their caregivers as caregivers tend to focus on their relative’s memory deficits as part of the diagnostic process.

There are also some limitations of the present study. First of all, while our expected dropout rate of 30% (Polak et al. 2017) could be nearly reached (here 36%), the expected conversion rate to dementia (*n* = 58 participants, of which *n* = 40 were expected to be patients with Alzheimer’s disease), was not observed in our study (we only had *n* = 5 participants with probable dementia). With 25 participants after 3 years reproducibly converting from normal cognitive state to MCI, however, we had a substantial number of subjects with deteriorating cognitive state. We previously reported (Haberstumpf et al. 2022b) that the dropout from baseline to the first follow-up after 3 years was modulated by cognitive impairments, with a bias into a healthier sample over time. We, therefore, used the “last observation carried forward” approach from visit 2 to visit 3, so that our results are influenced less by drop-out behaviour. Additionally, we extended our primary outcome parameter of MMSE sum score of < 24 to more liberal MMSE sum score < 27 and added the secondary outcome parameter of DemTect score < 13. In consequence, we were not able to calculate the prediction of Alzheimer’s disease, but to predict cognitive impairments. Although we were able to find significant predictive variables for cognitive decline over approximately eight years in the elderly, the total accuracy rate of 91.6% (with a specificity of 0.98 and a sensitivity of 0.33) is not suited for routine clinical application. This interpretation was further supported by the ROC analyses, which showed that our model has a sensitivity of 0.81 with a specificity of only 0.74.

However, our study underscores that both, VSEP and functional brain activity patterns, measure relevant factors in the development of cognitive impairments in the elderly.

We therefore propose that they should be included in further prediction studies with larger samples and multidimensional outcome parameters including the developing blood parameters.

Using quantitative rather than categorical variables may be more reliable for quantification of contributions from pathophysiological mechanisms and their spatial-temporal evolution (Baldacci et al. 2020). Given the complexity of the AD continuum, a set of multiple biomarkers will enhance diagnostic accuracy and characterize individual AD phenotypes (Gunes et al. 2022). Standardization of different methods used (Giangrande et al. 2023) as well as definition of international accepted cut-offs will be necessary (Lista et al. 2024). This may lead to a new era of personalized medicine, promising enhanced treatment initiation and monitoring with the potential for tailored interventions (Toader et al. 2023). Within this context, detecting the disease early is crucial because it enables interventions when treatments may be more effective (Grari et al. 2023; Lista et al. 2024; Hampel et al. 2022). Correct classification of AD likewise is crucial because patients without dementia falsely classified as having AD may nonetheless become candidates for passive immunotherapy, which may be harmful (Høilund-Carlsen et al. 2024).

To summarize, with the Vogel study we could show that VSEP and fNIRS measure relevant factors in the development of cognitive impairments in the elderly and thus should be included in further prediction studies with larger samples and multidimensional outcome parameters including the developing blood parameters.

## Data Availability

The data that support the findings of this study are not openly available due to reasons of sensitivity and are available from the corresponding author upon reasonable request.
